# Value of 3D MRI and Vaginal Opacification for the Diagnosis of Vaginal Endometriosis

**DOI:** 10.3389/fsurg.2020.614989

**Published:** 2020-12-18

**Authors:** Marc Bazot, Selma Beldjord, Lamia Jarboui, Clement Ferrier, Sofiane Bendifallah, Emile Daraï

**Affiliations:** ^1^Department of Radiology, Tenon University Hospital, Assistance Publique des Hôpitaux de Paris (AP-HP), Sorbonne University, Paris, France; ^2^Groupe de Recherche Clinique (GRC-6), Centre Expert En Endométriose (C3E), Assistance Publique Des Hôpitaux de Paris, Tenon University Hospital, Sorbonne Université, Paris, France; ^3^Centre Cardiologique du Nord, Saint Denis, France; ^4^Department of Gynaecology and Obstetrics, Tenon University Hospital, Assistance Publique des Hôpitaux de Paris (AP-HP), Sorbonne University, Paris, France; ^5^UMRS 938, Centre de recherche Saint Antoine, Faculté de Médecine, Sorbonne Université, Paris, France

**Keywords:** endometriosis, vaginal endometriosis, MRI, radiology, diagnosis

## Abstract

**Objective:** The aim of the study was to evaluate three-dimensional (3D) T2 MRI before and after vaginal opacification (VO) by gel (3DT2VO) and the additional value of 3DT1 with fat-suppression (3DT1FS) MRI in the diagnosis of vaginal endometriosis.

**Methods:** In this study conducted from 2010 to 2013, 51 patients scheduled for surgical treatment of endometriosis underwent MRI 1 day before surgery. Three readers (novice, intermediate, expert) were asked to retrospectively diagnose vaginal endometriosis independently and blindly using four different readings (i.e., 3DT2, 3DT2VO, 3DT2 with 3DT1FS, 3DT2VO with 3DT1FS). Vaginal endometriosis diagnosis was positive on observation of a thickening of vaginal walls on 3DT2 with or without high-signal-intensity spots on 3DT2 and/or 3DT1FS. The reference standard was surgery and histology. Descriptive analysis, Chi-square test, and ROC curves were used for statistical analysis.

**Results:** For all readers, the combination of 3DT2 and 3DT1FS significantly improved the diagnosis of vaginal endometriosis compared with 3DT2 (*p* = 0.002, *p* = 0.02, and *p* = 0.003). 3DT2VO significantly improved diagnosis for the intermediate reader (*p* = 0.01). High-signal-intensity spots on 3DT1FS had a sensitivity of 50–63.6%, specificity of 86.2–96.6%, and high positive likelihood ratios (14.5-Inf).

**Conclusion:** 3DT2 in association with 3DT1FS appears to be the best 3D MRI protocol for the diagnosis of vaginal endometriosis, whatever the level of experience of readers. The additional value of 3DT2VO is variable among the readers.

## Introduction

Deep pelvic endometriosis (DPE) is defined by the presence of fibrous/muscular infiltration of organs and anatomical structures containing endometrial like tissue below the peritoneum, regardless of the depth of infiltration (1). The most common locations of DPE include the torus uterinum, uterosacral ligaments, rectosigmoid colon, and vagina (2). Clinical examination can detect vaginal endometriosis but is limited for subtle vaginal lesions which are mainly linked to the presence of other DPE locations such as the torus uterinum and uterosacral ligaments (3).

Transvaginal sonography is the first-line technique for the diagnosis of endometriosis, allowing a dynamic examination, identifying trigger points for pain (4, 5). However, several meta-analyses have demonstrated that MRI is the best imaging technique to diagnose vaginal endometriosis even so no consensus exists regarding the optimal MRI protocol (1, 4–6). Multiplanar two-dimensional (2D) T2- in addition to T1-weighted MR sequences with fat-suppression are commonly performed with an accuracy varying from 71 to 90.8% (2, 3, 7–10). This heterogeneity could be partly explained by differences in MRI protocols and the criteria used to diagnose vaginal endometriosis.

Recent guidelines from the European society of Urogenital Radiology (ESUR) dedicated to MR imaging of pelvic endometriosis provide contradictory results about the value of vaginal opacification with gel (11). In addition, no specific data are available about the relevance of three-dimensional (3D) T2 and 3DT1 MRI sequences in this setting. These techniques are thus currently considered as an “option” in the diagnosis of vaginal endometriosis (11).

The aim of this study was to evaluate 3DT2 MRI before and after vaginal opacification (VO) by gel (3DT2VO) and the additional value of 3DT1 with fat-suppression (3DT1FS) MRI in the diagnosis of vaginal endometriosis.

## Patients and Methods

### Patients

The database of our pathological department was retrospectively analyzed to identify women who undergone surgery for pelvic endometriosis (*n* = 1005) on a study period from 2010 to 2013. The database of our radiological department was then reviewed to identify among these patients those who had MRI examinations. Patients having an MRI performed more than 6 months before surgery were offered another MRI the day before surgery. Exclusion criteria were: (i) patients <18 year-old, (ii) patients with previous surgery for DPE, (iii) patients with MRI performed 6 months before surgery and refusing the MRI the day before the surgery, and (iv) patients with DPE but without clear identification of vaginal endometriosis on histology even if the surgeon in the surgical report noted a partial colpectomy. Therefore, the final cohort included 51 non-consecutive symptomatic patients. All the patients were examined by two surgeons (ED, SB) with a high experience of pelvic endometriosis over 5 years. The first step consisted in a medical interrogation to evaluate symptoms (dysmenorrhea, deep dyspareunia, dysuria, dyschezia, and chronic pelvic pain). The second step corresponded to physical examination to assess vaginal endometriosis with speculum followed by vaginal digital examination to localize the various locations of endometriosis. Then rectal digitation examination was performed to evaluate rectal and parametrial involvement by DE. A systematic first-line transvaginal examination was systematically performed before MRI examination.

Institutional Ethics Committee approval was obtained and all patients gave their written informed consent to participate in the study (CEROG-2010-09).

### MRI Technique

MR images were acquired on a 1.5 Tesla system (General Electric HDTX, Milwaukee, USA) using a pelvic phased array. Patients fasted for 3 h and received an antispasmodic drug intravenously (Glucagen® Novo Nordisk, Paris, France) immediately before MR imaging to reduce bowel peristalsis. Systematic bowel cleaning was not performed.

A conventional protocol including sagittal, axial, coronal oblique 2DT2-weighted fast spin-echo (FSE) and 3DT1-weighted gradient-echo MR images with fat suppression (3DT1FS) was used. 3DT1FS MR images were acquired using SPoiled Gradient Recalling (SPGR) or Lava-Flex MRI sequences in 42 (82%) and 9 (18%) patients, respectively.

Three-dimensional imaging using coronal single slab 3D T2-weighted Fast-Spin-Echo (FSE) (called “CUBE”) was systematically added first without and then with vaginal opacification with 60 ml of sonographic gel. Neither rectal opacification nor gadolinium injection was performed in the present study. The parameters of the different MRI sequences are presented in Table 1.

**Table 1:** Parameters for 3DT2 and 3T1-weighted MR images with fat-suppression.

**Parameters**	**3DT2 Cube**	**3DT1 SPGR**	**3DT1 LAVA FLEX**
Repetition time (ms)/echo time (ms)	2,000/110	4/1.8	3.3/6.8
Field of view (mm)	300	300	300
Section thickness (mm)	1,4	2,4	2.4
Intersection gap (mm)	0	0	0
Acquisition plane	Coronal	Axial	Axial
Acquisition pixel size (mm)	1.2 × 1.3	1.2 × 1.2	0.9 × 0.9
Matrix	256 × 224	256 × 256	320 × 320
RVS (mm)	1.4 × 1.3 × 1.2	1.2 × 1.2 × 1.2	1.2 ×0.9 × 0.9
ETL	90	/	/
Bandwidth (Hz/pixel)	50	62	83
Parallel imaging	Yes	No	Yes
Imaging time	5 min 50	2 min 19	1 min 49

*RVS, Reconstructed voxel size; ETL, Echo train length*.

### MRI Analysis

Three radiologists with different degrees of experience in female pelvic MR imaging independently analyzed retrospectively the MR images. Reader 1 (SB) was a novice radiologist who had carried out 100 pelvic MR examinations using 3D TSE T2-weighted MR sequence over a 6-month period. Reader 2 (LJ) was a radiologist with 2 years of experience in gynecologic imaging. Reader 3 (MB) was a highly experienced radiologist (>25 years) in gynecologic imaging. All the readers were blinded to clinical findings and previous ultrasonographic or MR imaging results.

Each reader was asked to perform four readings to evaluate the presence or absence of vaginal endometriosis. First, they reviewed 3DT2 MR images without vaginal opacification. They went on to review the 3DT2 MR images with vaginal opacification (3DT2VO). Then they performed a combined analysis of MR images obtained on 3DT2 and 3DT1 with fat suppression (3DT1FS). Finally, they analyzed a combination of MR images obtained on 3DT2VO and 3DT1FS. Each MR analysis was performed using a review workstation with multiple reformations performed simultaneously. The time interval between readings was at least 15 days in order to minimize recall bias.

In the present study, the conventional MR imaging protocol (i.e., sagittal, axial, and coronal oblique 2DT2) was not taken into account for analysis.

### MR Imaging Criteria of Diagnosis

**Figure 1:**
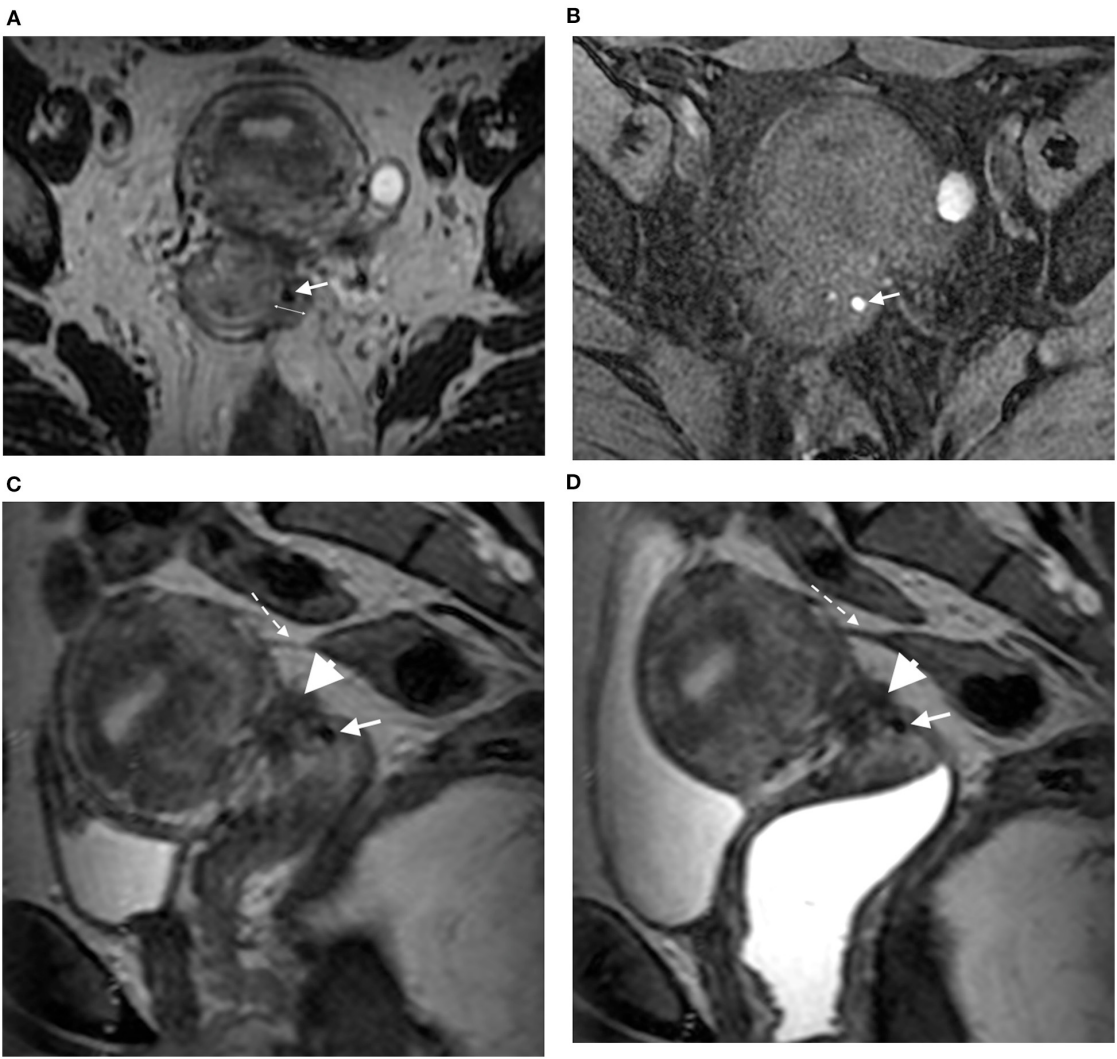
Axial 3DT2 **(A)** and 3DT1FS **(B)** MR images in 40-year-old woman show thickening of left lateral vaginal fornix (double arrow) containing spot (arrow) highly suggestive of vaginal endometriosis confirmed on surgery and histopathological examination. Sagittal 3DT2 **(C)** and 3DT2VO **(D)** MR images show associated torus uterinum involvement by DE (arrowhead) and rectosigmoid adhesion (dotted arrow). Note the absence of additional information provided by vaginal opacification.

Vaginal endometriosis was diagnosed in accordance with previously published criteria, i.e., thickening of the posterior and/or lateral vaginal wall on 3DT2 (Figure 1) with or without high-signal-intensity spots on 3DT1FS (2). The diagnosis was performed using a five-point scale to assign a confidence level for evaluating the absence or presence of vaginal endometriosis: 1, definitely absent; 2, probably absent; 3, indeterminate; 4, probably present; 5, definitely present.

**Figure 2:**
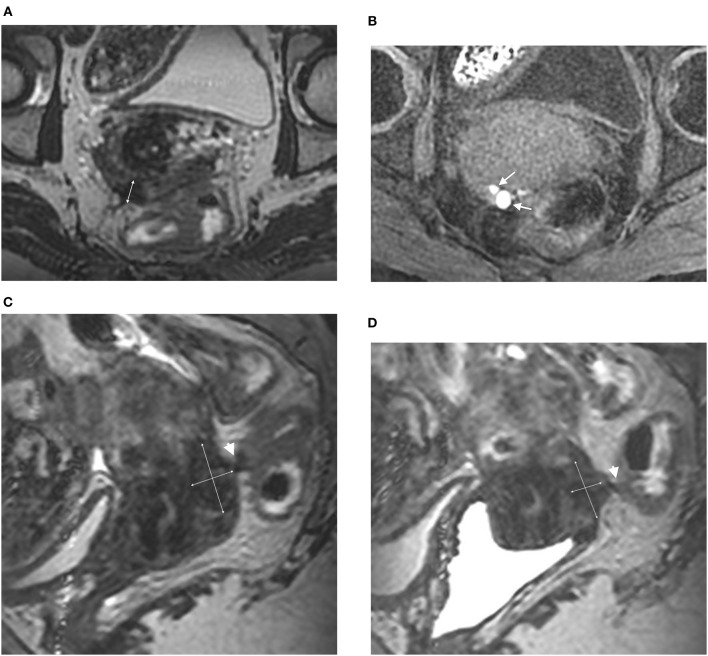
Axial 3DT2 **(A)** and 3DT1FS **(B)** MR images in 44-year-old woman show thickening of posterior vaginal wall (double arrow) containing multiple spots (arrows) highly suggestive of vaginal endometriosis confirmed on surgery and histopathological examination. Sagittal 3DT2 **(C)** and 3DT2VO **(D)** MR images confirm extensive thickening of posterior vaginal wall (double arrows) associated with rectal involvement by DE (arrowhead). Note the absence of additional information provided by vaginal opacification.

The diagnosis of other DPE locations was performed in accordance with previously described and validated MRI criteria (Figure 2) (2, 12). These locations were not specifically analyzed in the present study.

### Surgical and Histological Findings (The Reference Standard)

Vaginal endometriosis was diagnosed in one of the following isolated or associated circumstances:

- a) Endometrial like tissue (endometrial gland and stroma) found on histopathologic examination of a resected vaginal lesion (13).
- b) Direct visualization of a vaginal lesion of endometriosis on laparoscopy or laparotomy, with only fibrosis detected on histology (14), but with at least one other histologically proven location of endometriosis.

### Statistical Analysis

Confidence level ratings were used to calculate the sensitivity, specificity, positive, and negative predictive values, accuracy and positive (PLR) and negative likelihood ratios (NLR) [including 95% confidence interval (CI)] of MR images for each sequence in the diagnosis of vaginal endometriosis. Ratings of 1 or 2 indicated an absence of each location of DE and ratings of 4 or 5 indicated a presence. Ratings of 3 were considered to indicate an incorrect diagnosis.

A receiver operating characteristic (ROC) curve analysis and the area under the curve (AUC) was performed to compare the results of the different readings.

The intra- and interobserver agreement of the three readers for diagnosing vaginal endometriosis using the different MRI sequences were quantified by using weighted “statistics”; a *k*-value of <0.20 was considered to represent a poor agreement; 0.21–0.40, low agreement; 0.41–0.60, moderate agreement; 0.61–0.80, good agreement; and >0.80, excellent agreement. Specific evaluation of interobserver agreement of the three readers regarding the value of high signal intensity on 3DT1FS was added.

A *p* < 0.05 was considered to indicate a statistically significant association and all values were calculated using a two-tailed significance level. Statistical analysis and graphs were performed using MedCalc software (www.medcalc.be).

## Results

### Epidemiological, Surgical, and Histological Findings

Epidemiological characteristics of the study population were a median age of 34 years (range: 19–50) and body-mass index (BMI) of 22.9 kg/m^2^ (range: 14–43), respectively. The main symptoms were dysmenorrhea observed in 95% of cases, deep dyspareunia in all the patients, dyschezia in 70% of cases and infertility in 55% of cases. In addition to previous medical treatments, all the patients received GnRH analogs for 3 months before surgery.

Laparoscopy was performed in 48 of the 51 patients (94%) and laparotomy in three (6%). Vaginal endometriosis was diagnosed in 22 patients (43%).

Other locations such as ovarian, uterosacral ligaments, rectosigmoid colon, parametria, and bladder involvement by endometriosis were present in 35.3% (18/51), 96% (49/51), 76.4% (38/51), parametria (26/51), and bladder 5.6% (3/51), respectively.

### MRI Findings

The diagnosis of vaginal endometriosis obtained by each reader with the different readings (3DT2, 3DT2VO, 3DT2 and 3DT1FS, 3DT2VO and 3DT1FS) is provided in Table 2.

**Table 2:** Overall accuracy of MRI in the diagnosis of vaginal endometriosis per reader.

**Analysis**	**Reader 1**	**Reader 2**	**Reader 3**
**3DT2**			
Accuracy	60.8% (47.3–72.2)	68.6% (57–76)	80.4% (69.4–83.6)
Sensitivity	68.2% (52.5–81.4)	40.9% (27.5–49.4)	59.1% (46.3–62.8)
Specificity	55.2% (43.3–65.2)	89.7% (79.5–96.1)	96.6% (86.8–99.4)
PPV	53.6% (41.3–64)	75% (50.4–90.6)	92.9% (72.7–98.7)
NPV	69.6% (54.6–82.2)	66.7% (59.1–71.5)	75.7% (68.1–77.9)
PLR	1.52 (0.93–2.34)	3.95 (1.34–12.65)	17.14 (3.52–100.3)
NLR	0.58 (0.29–1.1)	0.66 (0.53–0.91)	0.42 (0.37–0.62)
**3DT2VO**			
Accuracy	66.7% (53.1–77.9)	80.4% (68.1–87.6)	88.2% (77.4–91.4)
Sensitivity	68.2% (52.5–81)	68.2% (53.9–76.5)	77.3% (64.7–81)
Specificity	65.5% (53.6–75.7)	89.7% (78.8–96)	96.6% (87–99.4)
PPV	60% (46.2–71.3)	83.3% (65.9–93.5)	94.4% (79.1–99)
NPV	73.1% (59.8–83.9)	78.8% (69.3–84.3)	84.8% (76.5–87.3)
PLR	1.97 (1.13–3.27)	6.6 (2.5–19)	22.4 (5–127.7)
NLR	0.48 (0.25–0.88)	0.36 (0.25–0.59)	0.24 (0.19–0.41)
**3DT2 and 3DT1FS**			
Accuracy	78.4% (66.2–84)	78.4% (66.1–85.7)	90.2% (79.1–95.4)
Sensitivity	90.9% (76.8–97.3)	63.6% (49.3–72)	90.9% (78.1–97)
Specificity	69% (58.2–73.8)	89.7% (78.8–96)	89.7% (79.9–94.2)
PPV	69% (58.2–73.8)	82.4% (63.9–93.2)	87% (74.7–92.7)
NPV	90.9% (76.8–97.3)	76.5% (67.2–81.9)	92.9% (82.8–97.8)
PLR	2.9 (1.8–3.7)	6.1 (2.3–18)	8.8 (3.8–16.8)
NLR	0.13 (0.04–0.4)	0.4 (0.3–0.6)	0.1 (0.03–0.27)
**3DT2VO and 3DT1FS**			
Accuracy	74.5% (62.2–80.1)	82.4% (70.1–89.5)	90.2% (79.1–95.4)
Sensitivity	90.9% (76.7–97.3)	72.7% (58.5–81)	90.9% (78.1–97)
Specificity	62.1% (51.3–67)	89.7% (78.9–95.9)	89.7% (79.9–94.2)
PPV	64.5% (54.4–69.1)	84.2% (67.8–93.7)	87% (74.7–92.7)
NPV	90% (74.4–97.1)	81.3% (71.5–86.9)	92.9% (82.8–97.8)
PLR	2.4 (1.5–2.9)	7 (2.8–19.7)	8.8 (3.8–16.8)
NLR	0.14 (0.04–0.45)	0.3 (0.2–0.5)	0.1 (0.03–0.27)

*Reading 1, 3DT2; Reading 2, 3DT2 with vaginal opacification; Reading 3, 3DT2 and 3DT1FS; Reading 4, 3DT2 and vaginal opacification and 3DT1FS*.

*PPV, positive predictive value; NPV, negative predictive value; PLR, Positive likelihood ratio; NLR, Negative likelihood ratio; 95% CI, 95% confidence interval*.

### Individual Reader Analysis

#### Reader 1

3DT2 MR analysis yielded a diagnosis of vaginal endometriosis in 28 of the 51 patients (54.9%). There were 13 false-positive results and seven false-negative results.

3DT2VO MR analysis yielded a diagnosis of vaginal endometriosis in 25 of the 51 patients (49%). There were 10 false-positive results and seven false-negative results.

3DT2 in association with 3DT1FS MR analysis yielded a diagnosis of vaginal endometriosis in 29 of the 51 patients (56.9%). There were nine false-positive results and two false-negative results.

3DT2VO in association with 3DT1FS MR analysis yielded a diagnosis of vaginal endometriosis in 31 of the 51 patients (60.7%). There were 11 false-positive results and two false-negative results.

#### Reader 2

3DT2 MR analysis yielded a diagnosis of vaginal endometriosis in 12 of the 51 patients (23.5%). There were three false-positive results and 13 false-negative results.

3DT2VO MR analysis yielded a diagnosis of vaginal endometriosis in 18 of the 51 patients (35.3%). There were three false-positive results and seven false-negative results.

3DT2 in association with 3DT1FS MR analysis yielded a diagnosis of vaginal endometriosis in 17 of the 51 patients (33.3%). There were three false-positive results and eight false-negative results.

3DT2VO in association with 3DT1FS MR analysis yielded a diagnosis of vaginal endometriosis in 19 of the 51 patients (37.3%). There were three false-positive results and six false-negative results.

#### Reader 3

3DT2 MR analysis yielded a diagnosis of vaginal endometriosis in 13 of the 51 patients (27.5%). There was one false-positive result and nine false-negative results.

3DT2VO MR analysis yielded a diagnosis of vaginal endometriosis in 17 of the 51 patients (36.3%). There was one false-positive result and five false-negative results.

3DT2 MR analysis in association with 3DT1FS MR analysis yielded a diagnosis of vaginal endometriosis in 20 of the 51 patients (41.2%). There were two false-positive results and three false-negative results.

3DT2VO MR analysis in association with 3DT1FS MR analysis yielded a diagnosis of vaginal endometriosis in 20 of the 51 patients (43.1%). There were three false-positive results and three false-negative results.

### Comparison of Different Readings

The relevance of the presence of high-signal-intensity spots detected by each reader on 3DT1FS is provided in Table 3. For all the readers, this MRI criterion was of low sensitivity (50–63.6%) and high specificity (86.2–96.6%). For the **Reader 2** and **Reader 3** it had a very high positive likelihood ratio (14.5-Inf).

**Table 3:** Value of high–signal–intensity spots on 3DT1FS in the diagnosis of vaginal endometriosis.

**MRI analysis**	**Reader 1**	**Reader 2**	**Reader 3**
**Reading 3**			
Accuracy	76.5% (63.8–85)	80.4% (70.7–80.4)	76.5% (64.8–82)
Sensitivity	63.6% (48.9–73.5)	54.5% (43.3–54.5)	54.5% (41–61)
Specificity	86.2% (75–93.7)	100% (91.5–100)	93.1% (82.8–98)
PPV	77.8% (59.8–89.8)	100% (79.4–100)	85.7% (64.4–95.8)
NPV	75.8% (65.9–82.3)	74.4% (68–74.4)	73% (64.9–76.8)
PLR	4.6 (1.9–11.6)	Inf	7.9 (2.4–30)
NLR	0.4 (0.28–0.68)	0.45 (0.45–0.62)	0.49 (0.4–0.7)
**Reading 4**			
Accuracy	76.5% (63.8–85)	76.5% (65.6–79.7)	80.4% (69.4–83.6)
Sensitivity	63.6% (48.9–73.5)	50% (37.4–53.7)	59.1% (46.3–62.8)
Specificity	86.2% (75–93.7)	96.6% (87–99.4)	96.6% (86.8–99.4)
PPV	77.8% (59.8–89.8)	91.7% (68.5–98.5)	92.9% (72.7–98.7)
NPV	75.8% (65.9–82.3)	71.8% (64.7–73.9)	75.7% (68.1–77.9)
PLR	4.6 (1.9–11.6)	14.5 (2.9–85.9)	17.1 (3.5–100.3)
NLR	0.4 (0.28–0.68)	0.5 (0.47–0.72)	0.42 (0.37–0.62)

*PPV, positive predictive value; NPV, negative predictive value; PLR, Positive likelihood ratio; NLR, Negative likelihood ratio; 95% CI, 95% confidence interval; 3DT1 FS, 3DT1 with fat-suppression technique*.

*Reading 3, 3DT2 with 3DT1FS; Reading 4, 3DT2 with vaginal opacification and with 3DT1FS*.

The ROC curve analyses are summarized in Table 4. For all readers, the combination of 3DT2 and 3DT1FS significantly improved the diagnosis of vaginal endometriosis in comparison to 3DT2 alone (0.002, 0.864, and 0.946 for the novice, intermediate, and experienced reader, respectively). 3DT2VO did not significantly improve diagnosis compared to 3DT2 alone for two of the three readers (0.4, 0.01, and 0.2, respectively). The most experienced reader in gynecological imaging obtained the best diagnostic confidence, whatever the MRI protocol used.

**Table 4:** Receiver Operating Characteristic (ROC) curve analysis.

**Analysis**	**Reader 1**	R1/R*	**Reader 2**	R1/R*	**Reader 3**	R1/R*
**3DT2**						
AUC (95% CI)	0.668 (0.52–0.79)		0.733 (0.59–0.84)		0.840 (0.7–0.92)	
SE	0.07		0.06		0.05	
*P*-value		NA		NA		NA
**3DT2VO**						
AUC (95% CI)	0.629 (0.48–0.76)		0.845 (0.71–0.93)		0.890 (0.77–0.96)	
SE	0.07		0.05		0.04	
*P*-value		0.4		0.01		0.2
**3DT2–T1FS**						
AUC (95% CI)	0.886 (0.79–0.95)		0.811 (0.67–0.9)		0.946 (0.84–0.99)	
SE	0.04		0.06		0.03	
*P*-value		0.002		0.02		0.003
**3DT2VO–T1FS**						
AUC (95% CI)	0.846 (0.71–0.93)		0.864 (0.73–0.94)		0.946 (0.84–0.99)	
SE	0.05		0.05		0.03	
*P*-value		0.03		0.004		0.003

R1, 3DT2; R2, 3DT2 with vaginal opacification (3DT2VO); R3, 3DT2 and 3DT1FS (3DT2-T1FS); R4, 3DT2 and vaginal opacification and 3DT1FS (3DT2VO-T1FS); AUC, Area under the curve; 95% CI interval, 95% Confidence Interval; R1 vs. R* where * corresponded to R2, R3, and R4, respectively; NA, not applicable.

Interobserver agreement according to the MRI protocol is presented in Table 5. A poor to moderate agreement was found between readers for 3DT2 without (kappa = 0.10–0.38) and 3DT2 with vaginal opacification (kappa = 0.28–0.57). A moderate agreement was found between readers for 3DT1FS associated with 3DT2 without (kappa = 0.54–0.58) and with vaginal opacification (kappa = 0.46–0.55).

**Table 5:** Interobserver agreement between readers according to MRI protocol.

**Vaginal endometriosis**	**Readers**		
**MRI protocol**	**Readers 1 and 2**	**Readers 1 and 3**	**Readers 2 and 3**
3DT2	*k* = 0,10	*k* = 0,17	*k* = 0,38
3DT2VO	*k* = 0,28	*k* = 0,28	*k* = 0,57
3DT2 with 3DT1FS	*k* = 0,55	*k* = 0,54	*k* = 0,58
3DT2VO with 3DT1FS	*k* = 0,51	*k* = 0,46	*k* = 0,55

*k, quadratic kappa; T1FS, T1 with fat-suppression technique*.

Intra-observer agreement of reader 1 (*k* = 1), 2 (*k* = 0.89), and 3 (*k* = 1) according to high-signal-intensity spots detected on readings 3 (3DT2 and 3DT1FS) and 4 (3DT2VO and 3DT1FS) was excellent.

Interobserver agreement for the detection of high-signal-intensity spots on 3DT1FS is displayed in Table 6. A good interobserver agreement (0.64–0.72) was found between readers in the detection of vaginal high-signal-intensity spots on 3DT1FS during reading 3. A moderate to good interobserver agreement (0.59–0.63) was found between readers in the detection of vaginal high-signal-intensity spots on 3DT1FS during reading 4.

**Table 6:** Interobserver agreement between readers according to high-signal-intensity spots detected on 3DT1FS.

**High-signal-intensity spots**	**Readers**		
**MRI protocol**	**Readers 1 and 2**	**Readers 1 and 3**	**Readers 2 and 3**
Reading 3	*k* = 0.72	*k* = 0.64	*k* = 0.69
Reading 4	*k* = 0.63	*k* = 0.59	*k* = 0.59

*k, quadratic kappa; 3DT1FS, 3DT1 with fat-suppression technique*.

*Reading 3, 3DT2 with 3DT1FS; Reading 4, 3DT2 with vaginal opacification and with 3DT1FS*.

## Discussion

The present study demonstrates that 3DT2 in association with 3DT1FS is the best MRI protocol for the diagnosis of vaginal endometriosis, whatever the level of experience of the reader. In contrast, the additional value of 3DT2VO varies depending on the reader.

Vaginal endometriosis is a frequent posterior DPE location, and is associated with dyschezia and deep dyspareunia (15, 16). Diagnosis of vaginal endometriosis is crucial before any surgical procedure for DPE. From an anatomical point of view, partial vaginal resection is associated with potential injury of the hypogastric nerve plexus located within the posterior and lateral vaginal walls (17). From a surgical point of view, the requirement of colpectomy exposes the patients to the risk of voiding dysfunction. Indeed, previous studies have underlined the relation between partial vaginal resection and bladder dysfunction requiring self-bladder catheterization (18, 19). Moreover, the concomitant resection of the vagina and rectosigmoid colon exposes the patient to a risk of rectovaginal fistula which, for many surgeons, justifies systematic defunctioning stoma although its relevance is a matter of debate (20).

Transvaginal sonography (TVS) is the first-line technique for the evaluation of pelvic endometriosis but its value for diagnosing vaginal endometriosis is controversial (3, 7, 21). Several meta-analyses underlined that MR imaging had a higher sensitivity (77%) than TVS (57–58%) for the diagnosis of vaginal endometriosis, with similar specificities (97–99%) (1, 5, 6). MR imaging is usually performed using sagittal, axial and coronal (± oblique) 2DT2- in addition to T1-weighted MR images without and with fat suppression for diagnosing pelvic endometriosis. Using this 2D MRI protocol, Nisenblat et al. found that diagnosis depended on the location and criteria used to diagnose endometriosis (1).

Various adjuncts have been tried since to improve the diagnostic performance of MRI for vaginal endometriosis, including vaginal gel opacification and 3DT2 MRI sequences. However, four studies which have evaluated the addition of vaginal gel opacification to the protocol give contradictory results (22–25). Chassang et al. reported an improvement in sensitivity between pre- and post-contrast 2DT2 MRI sequences; however, this improvement was only significant for junior radiologists but not for an expert (22). Bazot et al. did not find any significant difference with and without vaginal opacification, whatever the level of reader expertise (23). Fiaschetti et al. reported improved performance with TSE 2DT2 and 2DT1W MRI sequences (24). Finally, Uyttenhove et al. recently reported no significant difference, irrespective of reader experience (25).

The potential diagnostic value of 3DT2 MRI for pelvic endometriosis has been underlined by several authors (26, 27). These studies suggest that 3DT2 MR imaging provides acceptable image quality and improved 3D reconstructions in a shorter acquisition time and provides excellent visualization of the various locations (26, 27). However, the authors report variable sensitivities (60–80%) in the diagnosis of vaginal endometriosis, related to the level of expertise of the readers (26, 27).

Similarly to Saba et al., our results confirm that diagnostic confidence is a better indicator of the radiologist's interpretation to identify an imaging finding than the classical dichotomous choice (i.e., presence or absence) (10). In the ROC curve analysis, we found that the best performance was obtained by the most experienced reader in gynecological imaging whatever the MRI protocol used (10). In our study, 3DT2 without gel displayed a low AUC (0.67, 0.73, 0.84) for the three readers. These results are in agreement with those of Saba et al. reporting similar variable AUCs (0.69, 0.73, 0.81) for readers of different expertise when using 2DT2-weighted MRI (10). In the present study, 3DT2VO significantly increased the ability to detect vaginal endometriosis for the intermediate reader only. This finding suggests that it might be useful to design specific training for 3DT2 in association with vaginal opacification. In contrast, the best AUC (0.88, 0.81, 0.95, respectively) for all readers was obtained by using a combination of 3DT2 without gel and 3DT1FS. This could be explained by the additional value of high-signal-intensity spots detected by using 3DT1FS MR sequences. Specific analysis of high-signal-intensity spots for diagnosing deep endometriosis has rarely been reported independently (11). Our study suggests that this criterion has low sensitivity (50–64%) but relatively high specificity (86.2–96.6%) in the diagnosis of vaginal endometriosis. These data are in accordance with a preliminary report showing that nearly 61% of high-signal implants on T1 were visible in deep posterior endometriotic locations (2). This criterion was associated with a very high PLR (14.5-Inf) for intermediate and experienced readers underscoring its value in the setting of vaginal endometriosis. Furthermore, excellent intra-observer and a good interobserver agreement was found between readers in the detection of vaginal high-signal-intensity spots on 3DT1FS in association with 3DT2. Less agreement was found between readers in the detection of vaginal high-signal-intensity spots on 3DT1FS in association with 3DT2 with vaginal opacification. This could partly be explained by a decrease in the imaging quality observed with 3DT2 with vaginal opacification. Furthermore, we did not specifically evaluate gel instillation in the present study and Fiaschetti et al. observed that 10/83 patients (12%) refused the procedure or found it intolerable (24).

Several limitations of our study should be mentioned. Firstly, the prevalence of vaginal endometriosis in our population was very high (43%) in accordance with the design of our study. Secondly, a potential underestimation on histology of vaginal endometriosis could be link to pre-operative GnRH treatment related to the decrease of the glandular component. However, Netter et al. showed no significant difference on rectosigmoid nodule on MRI due to amenorrhea subsequent to pregnancy, GnRH analogs or contraceptive pill (28). Thirdly, all the patients were symptomatic and scheduled for surgery due to the presence of DPE which may have influenced the readers' interpretations. Thirdly, only one injection of antispasmodic drug was performed at the beginning of our MRI protocol owing to a poorer imaging quality of 3DT2VO and this represents another potential source of bias. Fourthly, all the patients received GnRH analogs that could have diminished the detection of high-signal-intensity spots within the vagina walls on 3DT1FS. Hence, an evaluation of high-signal-intensity spots within the vagina walls on 3DT1FS is required in the absence of GnRH analogs to evaluate the accuracy of this criterion. Finally, 3DT2VO was analyzed with 3DT1FS performed with different scout views. This could have induced some biases for the analysis of the location of high-signal-intensity spots on 3DT1FS and 3DT2-weighted MR imaging.

In conclusion, 3DT2 with 3DT1FS MR imaging is a valuable protocol for the evaluation of vaginal endometriosis. The systematic use of 3DT2VO is not supported by the present study.

## Data Availability Statement

The raw data supporting the conclusions of this article will be made available by the authors, without undue reservation.

## Ethics Statement

The studies involving human participants were reviewed and approved by CEROG-2010-09. Written informed consent for participation was not required for this study in accordance with the national legislation and the institutional requirements.

## Author Contributions

All authors participated in the collection of data, the statistical analysis, and the writing and correction of the manuscript.

## Conflict of Interest

The authors declare that the research was conducted in the absence of any commercial or financial relationships that could be construed as a potential conflict of interest.

## References

[1] Nisenblat V Bossuyt PM Farquhar C Johnson N Hull ML. Imaging modalities for the non-invasive diagnosis of endometriosis. Cochrane Database Syst Rev. (2016) 2:CD009591. 10.1002/14651858.CD009591.pub2 26919512 PMC7100540

[2] Bazot M Darai E Hourani R Thomassin I Cortez A Uzan S. Deep pelvic endometriosis: MR imaging for diagnosis and prediction of extension of disease. Radiology. (2004) 232:379–89. 10.1148/radiol.2322030762 15205479

[3] Bazot M Lafont C Rouzier R Roseau G Thomassin-Naggara I Darai E. Diagnostic accuracy of physical examination, transvaginal sonography, rectal endoscopic sonography, and magnetic resonance imaging to diagnose deep infiltrating endometriosis. Fertil Steril. (2009) 92:1825–33. 10.1016/j.fertnstert.2008.09.005 19019357

[4] Guerriero S Ajossa S Minguez JA Jurado M Mais V Melis GB. Accuracy of transvaginal ultrasound for diagnosis of deep endometriosis in uterosacral ligaments, rectovaginal septum, vagina and bladder: systematic review and meta-analysis. Ultrasound Obstet Gynecol. (2015) 46:534–45. 10.1002/uog.15667 26250349

[5] Guerriero S Saba L Pascual MA Ajossa S Rodriguez I Mais V. Transvaginal ultrasound vs magnetic resonance imaging for diagnosing deep infiltrating endometriosis: systematic review and meta-analysis. Ultrasound Obstet Gynecol. (2018) 51:586–95. 10.1002/uog.18961 29154402

[6] Noventa M Scioscia M Schincariol M Cavallin F Pontrelli G Virgilio B. Imaging modalities for diagnosis of deep pelvic endometriosis: comparison between trans-vaginal sonography, rectal endoscopy sonography and magnetic resonance imaging. A head-to-head meta-analysis. Diagnostics. (2019) 9:1–17. 10.3390/diagnostics9040225 31861142 PMC6963762

[7] Abrao MS Goncalves MO Dias JA Jr Podgaec S Chamie LP. Comparison between clinical examination, transvaginal sonography and magnetic resonance imaging for the diagnosis of deep endometriosis. Hum Reprod. (2007) 22:3092–7. 10.1093/humrep/dem187 17947378

[8] Chamie LP Blasbalg R Goncalves MO Carvalho FM Abrao MS de Oliveira IS. Accuracy of magnetic resonance imaging for diagnosis and preoperative assessment of deeply infiltrating endometriosis. Int J Gynaecol Obstet. (2009) 106:198–201. 10.1016/j.ijgo.2009.04.013 19467541

[9] Grasso RF Di Giacomo V Sedati P Sizzi O Florio G Faiella E. Diagnosis of deep infiltrating endometriosis: accuracy of magnetic resonance imaging and transvaginal 3D ultrasonography. Abdom Imaging. (2010) 35:716–25. 10.1007/s00261-009-9587-7 19924468

[10] Saba L Sulcis R Melis GB Ibba G Alcazar JL Piga M. Diagnostic confidence analysis in the magnetic resonance imaging of ovarian and deep endometriosis: comparison with surgical results. Eur Radiol. (2014) 24:335–43. 10.1007/s00330-013-3013-9 24026621

[11] Bazot M Bharwani N Huchon C Kinkel K Cunha TM Guerra A. European society of urogenital radiology (ESUR) guidelines: MR imaging of pelvic endometriosis. Eur Radiol. (2017) 27:2765–75. 10.1007/s00330-016-4673-z 27921160 PMC5486785

[12] Bazot M Jarboui L Ballester M Touboul C Thomassin-Naggara I Darai E. The value of MRI in assessing parametrial involvement in endometriosis. Hum Reprod. (2012) 27:2352–8. 10.1093/humrep/des211 22693170

[13] Cornillie FJ Oosterlynck D Lauweryns JM Koninckx PR. Deeply infiltrating pelvic endometriosis: histology and clinical significance. Fertil Steril. (1990) 53:978–83. 10.1016/S0015-0282(16)53570-5 2140994

[14] Adamson GD Nelson HP. Surgical treatment of endometriosis. Obstet Gynecol Clin North Am. (1997) 24:375–409. 10.1016/S0889-8545(05)70310-7 9163773

[15] Vercellini P Trespidi L De Giorgi O Cortesi I Parazzini F Crosignani PG. Endometriosis and pelvic pain: relation to disease stage and localization. Fertil Steril. (1996) 65:299–304. 10.1016/S0015-0282(16)58089-3 8566252

[16] Fauconnier A Chapron C Dubuisson JB Vieira M Dousset B Breart G. Relation between pain symptoms and the anatomic location of deep infiltrating endometriosis. Fertil Steril. (2002) 78:719–26. 10.1016/S0015-0282(02)03331-9 12372446

[17] Delmas A Appareil genital de la femme. In: Rouvière H, editor. Anatomie Humaine, Descriptive, Topographique et Fonctionnelle. Vol. 2 11 ed. Paris: Masson et Cie (1974). p. 597–651.

[18] Bonneau C Zilberman S Ballester M Thomin A Thomassin-Naggara I Bazot M. Incidence of pre- and postoperative urinary dysfunction associated with deep infiltrating endometriosis: relevance of urodynamic tests and therapeutic implications. Minerva Ginecol. (2013) 65:385–405. 24051939

[19] Zilberman S Ballester M Touboul C Chereau E Sebe P Bazot M. Partial colpectomy is a risk factor for urologic complications of colorectal resection for endometriosis. J Minim Invasive Gynecol. (2013) 20:49–55. 10.1016/j.jmig.2012.08.775 23131702

[20] Belghiti J Ballester M Zilberman S Thomin A Zacharopoulou C Bazot M. Role of protective defunctioning stoma in colorectal resection for endometriosis. J Minim Invasive Gynecol. (2014) 21:472–9. 10.1016/j.jmig.2013.12.094 24378832

[21] Hudelist G Ballard K English J Wright J Banerjee S Mastoroudes H. Transvaginal sonography vs. clinical examination in the preoperative diagnosis of deep infiltrating endometriosis. Ultrasound Obstet Gynecol. (2011) 37:480–7. 10.1002/uog.8935 21433168

[22] Chassang M Novellas S Bloch-Marcotte C Delotte J Toullalan O Bongain A. Utility of vaginal and rectal contrast medium in MRI for the detection of deep pelvic endometriosis. Eur Radiol. (2010) 20:1003–10. 10.1007/s00330-009-1627-8 19862535

[23] Bazot M Gasner A Lafont C Ballester M Darai E. Deep pelvic endometriosis: limited additional diagnostic value of postcontrast in comparison with conventional MR images. Eur J Radiol. (2011) 80:e331–9. 10.1016/j.ejrad.2010.12.006 21216125

[24] Fiaschetti V Crusco S Meschini A Cama V Di Vito L Marziali M. Deeply infiltrating endometriosis: evaluation of retro-cervical space on MRI after vaginal opacification. Eur J Radiol. (2012) 81:3638–45. 10.1016/j.ejrad.2011.06.058 21813257

[25] Uyttenhove F Langlois C Collinet P Rubod C Verpillat P Bigot J. Deep infiltrating endometriosis: Should rectal and vaginal opacification be systematically used in MR imaging? Gynecol Obstet Fertil. (2016) 44:322–8. 10.1016/j.gyobfe.2016.03.016 27216959

[26] Manganaro L Fierro F Tomei A Irimia D Lodise P Sergi ME. Feasibility of 3.0T pelvic MR imaging in the evaluation of endometriosis. Eur J Radiol. (2012) 81:1381–7. 10.1016/j.ejrad.2011.03.049 21497034

[27] Bazot M Stivalet A Darai E Coudray C Thomassin-Naggara I Poncelet E. Comparison of 3D and 2D FSE T2-weighted MRI in the diagnosis of deep pelvic endometriosis: preliminary results. Clin Radiol. (2013) 68:47–54. 10.1016/j.crad.2012.05.014 22832144

[28] Netter A d'Avout-Fourdinier P Agostini A Chanavaz-Lacheray I Lampika M Farella M. Progression of deep infiltrating rectosigmoid endometriotic nodules. Hum Reprod. (2019) 34:2144–52. 10.1093/humrep/dez188 31687764

